# Emerging Technologies to Promote and Evaluate Physical Activity: Cutting-Edge Research and Future Directions

**DOI:** 10.3389/fpubh.2014.00066

**Published:** 2014-06-27

**Authors:** Dan J. Graham, J. Aaron Hipp

**Affiliations:** ^1^Colorado School of Public Health and Department of Psychology, Colorado State University, Fort Collins, CO, USA; ^2^Prevention Research Center in St. Louis, Institute for Public Health, Brown School, Washington University in St. Louis, St. Louis, MO, USA

**Keywords:** physical activity, emerging technology, global positioning systems, accelerometers, smartphone app, built environment, online, interventions

Physical activity (PA) promotion and measurement efforts are achieving greater precision, ease of use, and scope by incorporating emerging technologies. This is significant for PA promotion because more precise measurement allows investigators to better understand where, when, and how PA occurs, thus enabling more effective targeting of particular behavior settings. Emerging technologies associated with measuring and evaluating PA are noteworthy because they can: (1) greatly increase external validity of measures and findings through ease of use and transferability; (2) significantly increase the ability to analyze patterns; (3) improve the ongoing, systematic collection and analysis of public health surveillance due to real-time capabilities; and (4) address the need for research about the cyber infrastructure required to cope with big data (multiple streams, aggregation, visualization, etc.). This special issue brings together a collection of the latest innovations and research on the application of technology to promote and measure PA, ranging from new video games aimed at children to measurement programs targeted at obese adults.

The first eight articles present emerging methodologies used to increase measurement specificity with regard to PA, the built environment, and geolocation. Combining multiple methodologies, Hurvitz et al. (1) provide a comprehensive overview of many emerging technologies including global positioning systems (GPS), accelerometers, smart phone applications (apps), and life logging. This article visually and systematically outlines the use of these emerging technologies and provides an exemplar case study. Use of GPS and accelerometers to track where and when PA occurs, as well as PA type, is covered by the next four manuscripts. Schipperijn et al. (2) test the accuracy of GPS in urban environments where building height, presence of water, and user speed and activity can each induce important GPS tracking errors. Testing various buffers around GPS-tracked bicycle trips, Madsen and colleagues (3) find that an elliptical buffer accounting for a participant’s home and clustered primary destinations is effective for data reduction and does not lose key built environment exposures. Ellis et al. (4) used machine learning to further investigate GPS and accelerometer measurement, error, and congruence across transportation user groups (pedestrians, cyclists, public transit, and personal vehicles). Finally, Klinker et al. (5) employed GPS and accelerometers to provide context to when and where children and adolescents are active outdoors.

Both Hirsch et al. (6) and Adlakha et al. (7) used public web feeds of GPS data to investigate where users of the MapMyRun app and website perform PA. Hirsch provides detailed background on the app and users, with a case study of activity in North Carolina. Adlakha examined the percentage of runs and walks mapped in St. Louis, MO, USA, parks and neighborhoods and how these varied by neighborhood socioeconomic status.

Kelly et al. (8) measured the built environment with Google StreetView© and reported the validity of using StreetView© in measuring the relationship between PA and built environment variations.

Several contributors to this special issue devised and tested technology-based interventions delivered in whole or in part through internet-enabled devices aimed at increasing PA, decreasing sedentary behavior, or both.

Dunton et al. (9) designed a smartphone app to monitor and enhance adolescent PA by supplementing users’ activity self-reports with objective data gathered through motion sensors already existent in smartphones. The use of existing technology inside apparatus many already own provides an affordable way for researchers to gather objective, real-time data. Further, apps like Dunton’s can help interpret PA data by combining them with user self-reports that shed light on contextual factors related to PA. Adams and colleagues (10) found that an intervention aiming to reduce sedentary behavior among obese women and incorporating both face-to-face and online components can reduce sedentary behavior and increase PA in this high-risk population. Lubans and colleagues (11) also developed a smartphone app aimed at promoting PA; this app additionally emphasized reduced screen-time and focused on low-income adolescent boys. These researchers identified key barriers to using PA-promotion smartphone apps that will likely be common across similar interventions and highlight lessons learned in incorporating various technologies into health promotion interventions. Maddison et al. (12) demonstrate that an intervention delivered through internet and phone can enhance PA among cardiac patients by increasing their self-efficacy. These studies underscore the benefits of combining technology (e.g., interventions delivered via computer or smartphone) and objective measurement (e.g., accelerometry, GPS) with subjective reports (e.g., psychological and social factors related to behaviors being assessed objectively).

An additional way to use smartphones for promoting PA is described by Barnett and colleagues (13). These authors discuss the growing field of active gaming among children, noting great potential to capitalize on moving active games outside of homes by using smartphones’ integrated features such as GPS, wireless internet, and camera. Synthesizing across these papers, excellent opportunities for measuring and promoting PA exist in interventions using smartphones, perhaps in conjunction with other technologies (e.g., online social networks), to facilitate active gaming in the real world.

Using technology to disseminate PA interventions enables PA advice to be distributed in a variety of ways. Alley et al. (14) using eye-tracking technology, demonstrate that online PA advice delivered via personally tailored videos better attracts and holds individuals’ attention than text-based messaging.

Finally, Graham and colleagues (15) designed a tool to promote PA among youth during classroom learning. *Jump In!* classroom response mats translate existing clicker technology used to collect real-time data from groups, such as students in classrooms, into a system whereby input is provided by jumping onto a mat, rather than finger pressing a handheld response box. In this case, as in all of the articles in this special issue, technology being used in a PA context already exists in another form or context, and it is the potential for this technology to be applied to PA promotion and measurement that is demonstrated and evaluated.

Undoubtedly, additional technologies currently used in other fields could be adopted for PA measurement and/or promotion. We look forward to creative uses of technology by PA researchers, building on the ways in which researchers such as those contributing to this special issue have incorporated emerging technologies into PA promotion and evaluation. Adopting useful technologies can increase the amount and quality of global recorded measurements of PA patterns and the potential to more effectively promote PA.

## Conflict of Interest Statement

The authors declare that the research was conducted in the absence of any commercial or financial relationships that could be construed as a potential conflict of interest.
